# On Security Performance of SWIPT Multi-User Jamming Based on Mixed RF/FSO Systems with Untrusted Relay

**DOI:** 10.3390/s24248203

**Published:** 2024-12-22

**Authors:** Xingyue Guo, Shan Tu, Dexian Yan, Yi Wang

**Affiliations:** 1 Key Laboratory of Electromagnetic Wave Information Technology and Metrology of Zhejiang Province, College of Information Engineering, China Jiliang University, Hangzhou 310018, China; p23030854013@cjlu.edu.cn (X.G.); 18a0303154@cjlu.edu.cn (D.Y.); 2 Guangxi Key Laboratory of Nuclear Physics and Nuclear Technology, Guangxi Normal University, Guilin 541001, China; ts@mailbox.gxnu.edu.cn

**Keywords:** RF/FSO, physical layer security, untrusted relay, multiuser interference, secrecy outage probability, average secrecy capacity

## Abstract

This paper presents research on the security performance of a multi-user interference-based mixed RF/FSO system based on SWIPT untrusted relay. In this work, the RF and FSO channels experience Nakagami-m fading distribution and Málaga (M) turbulence, respectively. Multiple users transmit messages to the destination with the help of multiple cooperating relays, one of which may become an untrusted relay as an insider attacker. In a multi-user network, SWIPT acts as a charging device for each user node. In order to prevent the untrusted relays from eavesdropping on the information, some users are randomly assigned to transmit artificial noise in order to interfere with untrusted relays, and the remaining users send information to relay nodes. Based on the above system model, the closed-form expressions of secrecy outage probability (SOP) and average secrecy capacity (ASC) for the mixed RF/FSO system are derived. The correctness of these expressions is verified by the Monte Carlo method. The influences of various key factors on the safety performance of the system are analyzed by simulations. The results show that the security performance of the system is considerably improved by increasing the signal–interference noise ratio, the number of interfering users, the time distribution factor and the energy conversion efficiency when the instantaneous signal-to-noise ratio (SNR) of the RF link instantaneous SNR is low.

## 1. Introduction

The rapid advances in wireless communication technology are gradually increasing the demand of wireless services. As a result, wireless spectrum resources are increasingly lacking. In addition, the low utilization of wireless spectrum resources exacerbates the problem of radio frequency (RF) spectrum scarcity. Compared with traditional RF communication, visible light communication technology has the advantages of rich spectrum resources, fast transmission speed, large bandwidth, excellent security and no electromagnetic radiation, and is being widely adopted to replace traditional radio frequency communication. However, the rate of visible light communication is usually low, generally hundreds of Mbps or less. This is mainly limited by the modulation speed of the LED light source and the sensitivity of the receiver [1]. Compared with visible light communication, the free-space optical (FSO) communication does not require a spectrum license, and has the advantages of high bandwidth, large transmission capacity, fast transmission rate and high security, which has attracted extensive attention from the academic community [2]. However, it is easily affected by various environmental factors, such as pointing errors and atmospheric turbulence, and is not suitable for long-distance communication [3].

In order to solve the above problems, a new communication model with unique advantages, the mixed RF/FSO system, is proposed [4,5]. Under severe weather conditions, this system combines the reliability of RF communication and the security of FSO communication, which can not only improve the overall performance of a communication system, but also effectively expands the communication distance. Due to the broadcast property of wireless RF channels in mixed systems, RF communication can be easily eavesdropped upon by malicious eavesdroppers for confidential information, which causes serious security risks in communication. Therefore, the physical layer security (PLS) performance of the mixed RF/FSO system is considered. As the PLS does not depend on any encryption algorithm and has low complexity, we only need to use the physical characteristics of wireless channels to achieve perfectly secure communication. Therefore, the PLS of the mixed RF/FSO system has received extensive attention.

So far, the mixed RF/FSO systems have studied the impacts of Channel Imperfection schemes [6], SWIPT [7] and multiple-input multiple-output (MIMO) [8] techniques on physical layer security. In recent years, the PLS of multi-user scheduling technology has been studied. Multi-user scheduling technology can improve the physical layer security of a wireless network without consuming extra resources, and has the advantage of expanding the communication range and enhancing the throughput of a communication system. For example, Ahmed et al. studied the security performance of multi-user single-input multiple-output (SIMO) RF/FSO relay networks with opportunistic user scheduling [9]. Yang et al. studied the introduction of a threshold-based multi-user scheduling method to achieve high communication security at the cost of low channel state information feedback [10]. Fan et al. studied physical layer security in multi-user relay networks, where multiple users communicate with base stations through multiple relays [11]. Gao et al. proposed a simultaneous wireless information and power transfer (SWIPT) energy-harvesting relay jamming-based mixed RF/FSO system, and studied its security performance optimization in the presence of an eavesdropper [12]. Compared with single-user communication, multi-user communication is more suitable for practical communication scenarios. Multi-user communication can not only improve the system throughput and the fault tolerance, but also significantly improves the reliability of the RF link. At present, multi-user scheduling technology is a popular topic in the PLS research of hybrid systems.

When single-user communication is blocked, multi-users can promptly call other users to complete the communication process. However, the existing multi-user systems also require a large number of relay nodes, which can be deployed in series or in parallel according to different topologies and network configurations. In order to improve the performance of the multi-user systems, a multi-relay scheme is proposed. The multi-relay scheme provides better spatial diversity and minimizes link loss at the cost of power allocation. It is an effective way to improve the stability and reliability of wireless communication networks. At present, there is little literature on the application of multi-relay communication technology in the PLS of mixed systems. Odeyemi et al. studied the security outage performance of partial relay selection in an AF mixed RF/FSO system with outdated channel state information [13]. Zhang et al. investigated the security performance of mixed RF/FSO systems with partial relay selection schemes under multi-aperture eavesdroppers [14]. Lei et al. considered that RF and FSO links experience Nakagami-m fading distribution and Malaga turbulence, respectively, and analyzed the impacts of SWIPT and multi-antenna techniques on safety performance [15].

However, the above documents are confidential transmissions to eavesdroppers, and do not consider the case of untrusted relays. In the public network, the relay may not have the same security clearance as the source–destination, and it will eavesdrop on the confidential information of the system, which threatens secure communication in the system. Therefore, it is necessary to consider the physical layer security of untrusted relays in a mixed RF/FSO system. At present, the PLS of the mixed RF/FSO system considering multi-user scheduling and multi-relay communication technologies under untrusted relay has not been reported.

Based on the above documents, this paper studies the security performance of a multi-user interference-based mixed RF/FSO system based on SWIPT untrusted relay. In this system, the RF links experience Nakagami-m fading distribution, and FSO links experience M fading distribution. We here consider a scene where a user node transmits information to a destination with the help of multiple relays, one of which may be corrupted to become an eavesdropper, which is named as the untrusted relay. Multiple wireless power supply jamming users are randomly assigned to interfere with the untrusted relay, and the rest of the wireless power supply users transmit information to the multi-relay nodes through maximum ratio merge (MRC). First, the cumulative distribution function (CDF) and probability density function (PDF) of the signal–interference noise ratio (SINR) of the untrusted relay under the action of multi-user jamming are derived. Second, the uniform CDF of the end-to-end signal-to-noise ratio (SNR) of the SIMO communication system under the DF relay scheme is calculated. Third, the approximate closed expressions of the system SOP and the ASC are further derived using the CDF based on the Meijer-G function and the generalized Gauss–Laguerre formula. Fourth, the accuracy of the derived expressions is verified by the Monte Carlo methods. Last, the effects of the average SINR of the interfering users, interfering user number, time distribution factor and energy conversion efficiency on the system security performance are analyzed by simulations.

## 2. System and Channel Model

**Figure 1:**
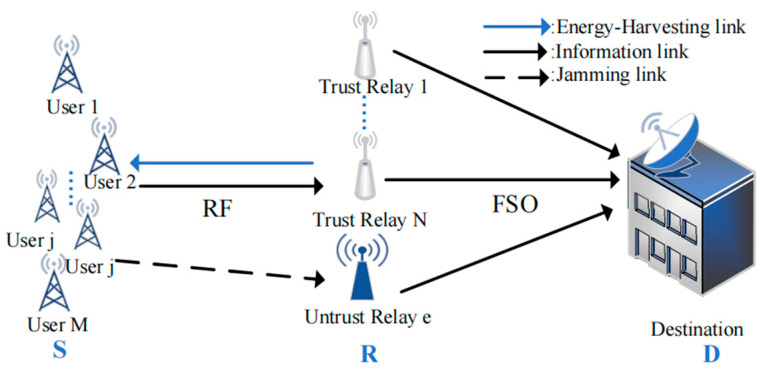
A SWIPT multi-user jamming-based mixed RF/FSO system.

As Figure 1 shows, a SWIPT multi-user jamming-based mixed RF/FSO system is considered. The system consists of multi-user nodes ($S_{m,j}(m,j\in \left\{1,\ldots ,M\right\})$), multiple single-antenna relays (R) and a single antenna target node (D). The RF link in the S-R segment and the FSO link in the R-D segment undergo Nakagami-m distribution and Málaga (M) distribution, respectively. The Nakagami-m distribution is a transformable fading model, which is widely used in the modeling of terrestrial mobile communication channels. It has strong applicability and can match experimental data well. The M distribution can effectively describe all atmospheric turbulence conditions from weak to strong, and it fits well with the experimental data. In the system, a relay is hacked as an eavesdropper and becomes an untrusted relay. In order to prevent the eavesdropping of untrusted relays, the user nodes randomly assign multiple wireless powered users as jammers to interfere with untrusted relay.

**Figure 2:**
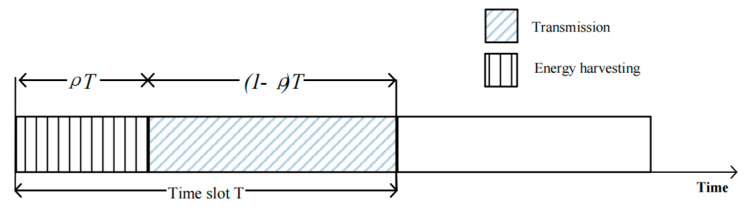
SWIPT time slot switching protocol structure.

Figure 2 shows the time slot switching protocol, which is used to design the total time block T for energy harvesting and transmitting interference signals. In the first time slot ρT, the user node S obtains energy from the electrical signal of each secondary node R and stores it, and the collected energy is used for information transmission in the second time slot (1−ρ)T. According to the energy collected by each node, the transmit power used by each user node to transmit information in the second time slot is obtained. Multiple jamming user nodes are randomly assigned, and the instantaneous channel state information of each user is used to jam the untrusted relay, while the remaining users pass the information to the relay node R through the maximum proportional combination (MRC). The destination node D then receives the signal from each relay node using the equal-gain combining (EGC) method.

In the energy harvesting stage, the relay node R first sends a signal to the user node i, which is composed of the signal itself and the noise. The signal received by each user node is as follows:

(1) $$y_{Ri}=\frac{1}{\sqrt{d_{Ri}^{\tau }}}(\sqrt{P_{R}}h_{Ri}x_{Ri}+n_{R})$$

where i∈{m,j}, j represents each interfering user node, and m represents each transmitting user node. $P_{R}$ is the transmitting power of each relay node. $x_{Ri}$ is the normalized signal of relay node, and the signal will be affected by the channel in the transmission process, while the channel coefficient between the relay node R and each user node is $h_{Ri}$. This coefficient indicates that the signal may be strengthened or weakened as it moves from the secondary node to the user node. The signal will also decay with the increase in distance in the transmission process; the distance between each relay node and each user node is $d_{Ri}$. τ is the path loss exponent, so the signal strength is inversely proportional to the square root of the distance, which is $\frac{1}{\sqrt{d_{Ri}}}$. Finally, the signal is disturbed by additive Gaussian white noise $n_{R}$ with zero mean and variance $\sigma_{SR}^{2}$ at the relay node.

According to (1), without considering noise, the energy obtained by each user node from each relay node is expressed as [16]:

(2) $$E_{i}=\eta \rho \frac{1}{d_{Ri}}P_{R}h_{Ri}^{2}T$$

where η is the energy conversion efficiency, indicating the efficiency of converting the RF signal to direct current (DC) by multiple users; ρ is the time switching coefficient, representing the proportion of time switching between the energy collection stage and the information transmission stage. The time period is T, and the energy of the signal during transmission is inversely proportional to the distance, that is, $\frac{1}{d_{Ri}}$.

In the second time slot (1−ρ)T, the transmission power of each user and node to transmit information is expressed as

(3) $$P_{m}=\frac{\eta \rho P_{R}{\left|h_{Rm}\right|}^{2}}{(1-\rho )d_{Rm}}$$

where (1−ρ) represents the time proportion of the information transmission stage, and ρ represents the time proportion of the energy collection stage.

The power used by the friendly interference user node to transmit the interference signal is expressed as

(4) $$P_{j}=\frac{\eta \rho P_{R}{\left|h_{Rj}\right|}^{2}}{(1-\rho )d_{Rj}}$$

Once each user node accumulates energy after the charging stage, the interference signal will cause certain interference in the relay node of a single user. Using the artificial interference generation method given in [17], it can be gathered that the jammer generates an $S_{j}\times (S_{j}-1)$ matrix W for multiple interfering users, which is an orthonormal basis of the null space of $h_{Rj}$, It also generates a vector **v** with ${(S}_{j}-1)$ independent identically distributed complex Gaussian random elements with a normalized variance. Subsequently, the interfering users transmit Wv as the jamming signal. The signal received by the relay node R at this time is as follows:

(5) $$y_{SR}=\left\{\begin{array}{ll} \frac{\sqrt{P_{S}}}{\sqrt{d_{mR}^{\tau }}}\sum_{m=1}^{M}h_{mR}x_{\mathrm{mR}}+\frac{\sqrt{P_{J}}}{\sqrt{d_{JR}^{\tau }}}h_{JR}x_{JR}+e_{R}, & {\;S}_{j}=1\\ \frac{\sqrt{P_{S}}}{\sqrt{d_{mR}^{\tau }}}\sum_{m=1}^{M}h_{mR}x_{\mathrm{mR}}+e_{R}, & {\;S}_{j}>1 \end{array}\right.$$

where, when the number of interfering user nodes $S_{J}=1$, the transmitting power of the source node is $P_{S}$, the normalized signal sent by the user node is $x_{mR}$, the channel coefficient is $h_{mR}$, and the distance is $d_{mR}$. The transmitted power of the friendly interference user node is $P_{J}$, the normalized signal sent is $x_{JR}$, the channel coefficient is $h_{JR}$, and the distance is $d_{JR}$. The additive white Gaussian noise at the relay node is $e_{R}$, with zero mean and variance $\sigma_{R}^{2}$. When $S_{j}>1$, the interference signal is transmitted to the null space of the $h_{JR}$, so the signal received at the trusted relay is not affected by the interference user. Based on the above analysis, the following scenarios for the number of interfering users are all based on $S_{j}>1$.

Under the action of the jamming user, the signal received by the untrusted relay node E can be expressed as:

(6) $$y_{SE}=\frac{\sqrt{P_{m}}}{\sqrt{d_{mE}^{\tau }}}\sum_{m=1}^{M}h_{\mathrm{mE}}x_{mR}+\frac{\sqrt{P_{j}}}{\sqrt{d_{JE}^{\tau }}}\sum_{j=1}^{J}h_{\mathrm{JE}}\frac{Wv}{\sqrt{S_{j}-1}}+n_{E}$$

where $h_{mE}$ is the channel coefficient between each user node m and the untrusted relay, $h_{JE}$ is the channel coefficient between the interfering user j node and the untrusted relay, and $n_{E}$ is the additive Gaussian white noise with zero mean and $\sigma_{E}^{2}$ variance at the untrusted relay.

### 2.1. RF Channel Model

The interference signal sent by the interfering users will only affect the untrusted relay. In the communication scene, the SINR of the untrusted relay is

(7) $$\gamma_{E}=\frac{\gamma_{SE}}{\gamma_{JE}+1}$$

where $\gamma_{\mathrm{JE}}=\frac{P_{R}{\left\|\sum_{j=1}^{J}h_{\mathrm{JE}}W\right\|}^{2}}{\sigma_{E}^{2}d_{\mathrm{JE}}^{\tau }(S_{J}-1)}=\frac{\rho \eta P_{R}S_{J}{\left\|\sum_{j=1}^{J}h_{\mathrm{JE}}W\right\|}^{2}{\left|h_{\mathrm{RJ}}\right|}^{2}}{d_{\mathrm{RJ}}^{\tau }d_{\mathrm{JE}}^{\tau }{(1-\rho )N}_{0}(S_{J}-1)}$, $\gamma_{\mathrm{SE}}=\frac{\rho \eta P_{R}{\left|h_{RE}\right|}^{2}}{{(1-\rho )\sigma }_{E}^{2}d_{\mathrm{Ri}}^{\tau }d_{\mathrm{iE}}^{\tau }}$.

All RF links between S and R undergo Nakagami-m fading distribution, and the PDF and CDF of the instantaneous SNR $\gamma_{k}$ of RF links can be written as [15]

(8) $$f_{\gamma_{k}}(\gamma )=\frac{1}{\Gamma (m_{k}N_{k})}{\left(\frac{m_{k}}{\Omega_{k}}\right)}^{m_{k}N_{k}}\gamma_{k}^{m_{k}N_{k}-1}\exp(-\frac{m_{k}}{\Omega_{k}}\gamma_{k})$$

(9) $$F_{\gamma_{k}}(\gamma )=1-\exp(-\frac{m_{k}}{\Omega_{k}}\gamma_{k})\sum_{t=0}^{m_{k}N_{k}-1}\frac{1}{t!}{\left(\frac{m_{k}}{\Omega_{k}}\gamma_{k}\right)}^{t}$$

where k∈{SR,SE,JE}, $\Omega_{SR}=\frac{\rho \eta P_{R}\lambda_{SR}}{(1-\rho )\sigma_{R}^{2}d_{SR}^{\iota }}$, $\Omega_{SE}=\frac{\rho \eta P_{R}\lambda_{SE}}{(1-\rho )\sigma_{E}^{2}d_{SE}^{\iota }}$ and $\Omega_{JE}=\frac{\rho \eta P_{R}{S_{J}\lambda }_{JE}}{(1-\rho )\sigma_{E}^{2}{d_{SR}^{\iota }d}_{JE}^{\iota }}$ denote the average power channel gains of each channel, respectively. $N_{k}$ represents the number of antennas for each channel.

The cumulative distribution function (CDF) of the instantaneous signal-to-noise ratio for the link with the worst user interference eavesdropper at the transmitting end is

(10) $$F_{\gamma_{S_{J}E_{1}}}(\gamma )=1-{\left[\exp\left(-\frac{m_{S_{J}E_{1}}}{\Omega_{S_{J}E_{1}}}\gamma \right)\sum_{a=0}^{m_{S_{J}E_{1}}N_{S_{J}E_{1}}-1}\frac{1}{a!}{\left(\frac{m_{S_{J}E_{1}}}{\Omega_{S_{J}E_{1}}}\gamma \right)}^{a}\right]}^{n}$$

Among them, $\Omega_{S_{J}E_{1}}=\frac{P_{S_{J}}\lambda_{S_{J}E_{1}}}{\sigma_{S_{J}E_{1}}^{2}d_{S_{J}E_{1}}^{\tau }}$ is the average signal-to-noise ratio of the link for the worst user interference eavesdropper.

Using (7), (8), (9), and (10), the CDF of instantaneous SINR $\gamma_{SJE}$ is obtained as follows [18]:

(11) $$\begin{array}{l} F_{\gamma_{SJE}}(\gamma )\;=1-\sum_{i=0}^{m_{SE}-1}\frac{1}{t!}{\left[\frac{m_{SE}}{\Omega_{SE}(S_{j}-1)}\right]}^{t}\frac{1}{\Gamma (m_{JE}(S_{j}-1))}\sum_{k=0}^{t}\left(\begin{array}{c} t\\ k \end{array}\right){\left(\frac{\Omega_{JE}}{m_{JE}}\right)}^{k}\gamma^{t}\\ \times \exp\left[-\frac{m_{SE}}{\Omega_{SE}(S_{j}-1)}\gamma \right]G\begin{array}{c} 1,1\\ 1,1 \end{array}\left[\frac{m_{SE}\Omega_{JE}}{\Omega_{SE}(S_{j}-1)}\gamma \left|\begin{array}{c} 1-k-m_{JE}(S_{j}-1)\\ 0 \end{array}\right.\right] \end{array}$$

The derivative operation is performed on the CDF represented by (11). The corresponding PDF is obtained,

(12) $$\begin{array}{l} f_{\gamma_{SJE}}(\gamma )\;=\frac{1}{\Gamma (m_{SE})\Gamma (m_{JE}(S_{j}-1))}{\left[\frac{m_{SE}}{\Omega_{SE}(S_{j}-1)}\right]}^{m_{SE}}\sum_{k=0}^{m_{SE}-1}\left(\begin{array}{c} m_{SE}-1\\ k \end{array}\right){\left(\frac{\Omega_{JE}}{m_{JE}}\right)}^{k}\\ \times \gamma^{m_{SE}-1}\exp\left[-\frac{m_{SE}}{\Omega_{SE}(S_{j}-1)}\gamma \right]G\begin{array}{c} 1,1\\ 1,1 \end{array}\left[\frac{m_{SE}\Omega_{JE}}{\Omega_{SE}m_{JE}(S_{j}-1)}\gamma \left|\begin{array}{c} 1-k-m_{JE}(S_{j}-1)\\ 0 \end{array}\right.\right] \end{array}$$

### 2.2. FSO Channel Model

Each relay node converts the received RF signal into an FSO signal through subcarrier intensity modulation, and the destination node D receives the FSO signal in EGC. Therefore, the PDF and CDF of the FSO fading channel are expressed as follows [19]:

(13) $$\begin{array}{ll} f_{\gamma_{RD}}\left(\gamma \right) & =\frac{1}{2}{\sqrt{\gamma_{RD}\bar{\gamma_{RD}}}}^{-\frac{1}{2}}A\sum_{K=1}^{N(\beta -1)}a_{m}(\sqrt{\frac{\gamma_{RD}}{\bar{\gamma_{RD}}}}{)}^{\frac{N\alpha +N+K}{2}-1}\\ \; & \times K_{N\alpha -N-K}(2\sqrt{\frac{N\alpha \beta }{g\beta +\Omega^{\prime }}}) \end{array}$$

(14) $$\begin{array}{ll} F_{\gamma_{RD}}\left(\gamma \right) & =\frac{A}{2}\sum_{k=0}^{N(\beta -1)}a_{k}{\left(\frac{\gamma_{RD}}{\bar{\gamma_{RD}}}\right)}^{\frac{N\alpha +N+K}{4}}\\ \; & \times G_{1,3}^{2,1}\left[\frac{N\alpha \beta }{g\beta +\Omega^{\prime }}\sqrt{\frac{\gamma_{RD}}{\bar{\gamma_{RD}}}}\left|\begin{array}{l} k_{1}\\ k_{2} \end{array}\right.\right] \end{array}$$

where $k_{1}=1-\frac{N\alpha +N+k}{2}$, $k_{2}=(\frac{N\alpha -N-k}{2},\frac{-N\alpha +N+k}{2},\frac{-N\alpha -N-k}{2}$), $A={\left(\frac{N\alpha \beta }{g\beta +\Omega^{\prime }}\right)}^{N\beta }\frac{2{\left(N\alpha \right)}^{N\alpha }}{g^{N}{\left(N\beta \right)}_{N-1}\Gamma (N\alpha )}$, αk=Nβ−1Nβ−1−k1k!Ω′gβ+Ω′g1gk×(β(gβ+Ω′)Nα)Nα−N−k2, ${\bar{\gamma }}_{RD}$ is the average SNR of the FSO link, g represents the average power of the classical scattering component $U_{S}^{G}$, $\Omega^{\prime }$ represents the average power of the coherent contribution, α is a positive parameter regarding the effective quantity of large-scale units in the scattering process, and β is a natural number related to the diffraction effect produced by small-scale eddies. 

## 3. End-to-End SNR Statistics

Under DF relay, the end-to-end instantaneous SNR is expressed as

(15) $$\gamma_{\mathrm{SRD}}=\frac{\gamma_{SR}\gamma_{RD}}{\gamma_{SR}+\gamma_{RD}+1}≅\min(\gamma_{SR},\gamma_{RD})$$

The end-to-end CDF of the joint channel is

(16) $$\begin{array}{ll} F_{\gamma_{SRD}}(\gamma ) & =P_{r}[\min(\gamma_{SR},\gamma_{RD})<\gamma ]\\ \; & =F_{\gamma_{SR}}(\gamma )+F_{\gamma_{RD}}(\gamma )-F_{\gamma_{SR}}(\gamma )F_{\gamma_{RD}}(\gamma ) \end{array}$$

Substituting (9) and (13) into (15) yields

(17) $$\begin{array}{l} F_{\gamma_{SRD}}(\gamma )\;=1-\exp(-\frac{m_{k}}{\Omega_{k}}\gamma_{k})\sum_{t=0}^{m_{k}N_{k}-1}\frac{1}{t!}{\left(\frac{m_{k}}{\Omega_{k}}\gamma_{k}\right)}^{t}\\ +\exp(-\frac{m_{k}}{\Omega_{k}}\gamma_{k})\sum_{t=0}^{m_{k}N_{k}-1}\frac{1}{t!}{\left(\frac{m_{k}}{\Omega_{k}}\gamma_{k}\right)}^{t}\frac{A}{2}\\ \times \sum_{k=0}^{N(\beta -1)}a_{k}{\left(\frac{\gamma }{\bar{\gamma_{RD}}}\right)}^{\frac{N\alpha +N+K}{4}}G_{1,3}^{2,1}\left[\frac{N\alpha \beta }{g\beta +\Omega^{\prime }}\sqrt{\frac{\gamma }{\bar{\gamma_{RD}}}}\left|\begin{array}{l} k_{1}\\ k_{2} \end{array}\right.\right] \end{array}$$

## 4. Secrecy Outage Probability Analysis

SOP is one of the security benchmarks, defined as a probability event that will occur when the instantaneous security capacity falls below the target safety rate $R_{S}$. Therefore, the SOP lower bound expression for the mixed system [20] is as follows:

(18) $$P_{out}^{L}(R_{s})=\int_{0}^{\infty }F_{\gamma_{SRD}}(\theta \gamma )f_{\gamma_{SE}}(\gamma )d\gamma$$

where $\theta =exp(R_{S})$. Substituting (12) and (17) into (18), the end-to-end SOP can be written as

(19) $$P_{\gamma_{SRD}}(R_{s})\;≜=\Lambda_{1}-\Lambda_{2}+\Lambda_{3}$$

(20) $$\begin{array}{ll} \Lambda_{1}= & \frac{1}{\Gamma (m_{E})\Gamma (m_{JE}(S_{j}-1))}{\left[\frac{m_{E}}{{\bar{\gamma }}_{SE}(S_{j}-1)}\right]}^{m_{E}}\sum_{k=0}^{m_{E}}\left(\begin{array}{c} m_{E}\\ k \end{array}\right){\left(\frac{m_{JE}}{\Omega_{JE}}\right)}^{-k}\\ & \times \int_{0}^{\infty }\begin{array}{l} \gamma^{m_{E}-1}\exp\left[-\frac{m_{E}}{\Omega_{SE}(S_{j}-1)}\gamma \right]\\ \times G\begin{array}{c} 1,1\\ 1,1 \end{array}\left[\frac{m_{E}\Omega_{JE}}{\Omega_{SE}m_{JE}(S_{j}-1)}\gamma \left|\begin{array}{c} 1-k-m_{JE}(S_{j}-1)\\ 0 \end{array}\right.\right] \end{array}d\gamma \end{array}$$

(21) $$\begin{array}{l} \Lambda_{2}=\sum_{t=0}^{N_{R}m_{SR}-1}\frac{1}{t!}{\left(\frac{m_{SR}\theta }{\Omega_{SR}}\right)}^{t}\frac{1}{\Gamma (m_{E})\Gamma (m_{RE}(S_{j}-1))}{\left[\frac{m_{SE}}{\Omega_{SE}(S_{j}-1)}\right]}^{m_{SE}}\\ \times \sum_{k=0}^{m_{SE}}\left(\begin{array}{c} m_{SE}\\ k \end{array}\right){\left(\frac{m_{JE}}{\Omega_{JE}}\right)}^{k}\int_{0}^{\infty }\begin{array}{l} \gamma^{t+m_{E}-1}\exp\left[-\left(\frac{m_{SR}\theta }{\Omega_{SR}}+\frac{m_{SE}}{\Omega_{SE}(S_{j}-1)}\right)\gamma \right]\\ G\begin{array}{c} 1,1\\ 1,1 \end{array}\left[\frac{m_{SE}\Omega_{JE}}{\Omega_{SE}m_{JE}(S_{j}-1)}\gamma \left|\begin{array}{c} 1-k-m_{JE}(S_{j}-1)\\ 0 \end{array}\right.\right] \end{array}d\gamma \end{array}$$

(22) $$\begin{array}{l} \Lambda_{3}=\sum_{t=0}^{N_{R}m_{SR}-1}\frac{1}{t!}{\left(\frac{m_{SR}\theta }{\Omega_{SR}}\right)}^{t}\frac{1}{\Gamma (m_{SE})\Gamma (m_{JE}{(}_{J}-1))}{\left[\frac{m_{SE}}{\Omega_{SE}(N_{J}-1)}\right]}^{m_{SE}}\sum_{k=0}^{m_{SE}}\left(\begin{array}{c} m_{SE}\\ k \end{array}\right)\\ \times {\left(\frac{m_{JE}}{\Omega_{JE}}\right)}^{k}\int_{0}^{\infty }\begin{array}{l} \gamma^{t+m_{SE}-1}\exp\left[-\left(\frac{m_{SR}\theta }{\Omega_{SR}}+\frac{m_{SE}}{\Omega_{SE}(S_{j}-1)}\right)\gamma \right]\\ \times G\begin{array}{c} 1,1\\ 1,1 \end{array}\left[\frac{m_{SE}\Omega_{JE}}{\Omega_{SE}m_{JE}(S_{j}-1)}\gamma \left|\begin{array}{c} 1-k-m_{JE}(S_{j}-1)\\ 0 \end{array}\right.\right] \end{array}\\ \times \frac{A}{2}\sum_{k=0}^{N(\beta -1)}a_{k}{\left(\frac{\gamma }{\bar{\gamma_{RD}}}\right)}^{\frac{N\alpha +N+K}{4}}G_{1,3}^{2,1}\left[\frac{N\alpha \beta }{g\beta +\Omega^{\prime }}\sqrt{\frac{\theta \gamma }{\bar{\gamma_{RD}}}}\left|\begin{array}{l} k_{1}\\ k_{2} \end{array}\right.\right]d\gamma \end{array}$$

By applying the integral constancy equation given in [21], the exponential function is converted to Meijer-G. Then, using the integral formula of the Meijer-G function given in [22] and substituting (20)–(22) into (19), followed by some mathematical simplifications using the above calculation method, the end-to-end SOP can be shown as follows:

(23) $$\begin{array}{l} P_{\gamma_{SRD}}(R_{s})=\frac{1}{\Gamma (m_{SE})\Gamma (m_{SR}(S_{j}-1))}\sum_{k=0}^{m_{SE}-1}\left(\begin{array}{c} m_{SE}-1\\ k \end{array}\right){\left(\frac{\Omega_{JE}}{m_{JE}}\right)}^{k}\\ \times G\begin{array}{c} 1,2\\ 2,1 \end{array}\left[\frac{\Omega_{JE}}{m_{JE}}\left|\begin{array}{c} 1-k-m_{JE}(S_{j}-1),1-m_{SE}\\ 0 \end{array}\right.\right]\\ -\sum_{t=0}^{N_{S}m_{SR}-1}\frac{1}{t!}{\left(\frac{m_{SR}\theta }{\Omega_{SR}}\right)}^{t}\frac{1}{\Gamma (m_{SE})\Gamma (m_{SR}(S_{j}-1))}{\left[\frac{m_{SE}}{\Omega_{SE}(S_{j}-1)}\right]}^{m_{SE}}\sum_{k=0}^{m_{SE}}\left(\begin{array}{c} m_{SE}\\ k \end{array}\right){\left(\frac{\Omega_{JE}}{m_{JE}}\right)}^{k}\\ \times \left(\begin{array}{l} {\left[\frac{m_{SR}\theta (S_{j}-1)\Omega_{SE}+m_{SE}\Omega_{SR}}{\Omega_{SE}\Omega_{SR}(S_{j}-1)}\right]}^{-m_{SE}-t}\\ G\begin{array}{c} 1,2\\ 2,1 \end{array}\left[\frac{\Omega_{SE}\Omega_{SR}m_{SE}}{(m_{SR}\theta (S_{j}-1)\Omega_{SE}+m_{SE}\Omega_{SR})m_{JE}}\left|\begin{array}{c} 1-k-m_{JE}(S_{j}-1),1-m_{SE}\\ 0 \end{array}\right.\right]\\ -\sum_{j=1}^{t}H_{j}\gamma_{j}^{t+m_{SE}-0.5}\exp\left[-\left(\frac{m_{SR}\theta }{\Omega_{SR}}+\frac{m_{SE}}{\Omega_{SE}(S_{j}-1)}-1\right)\gamma_{j}\right]\\ \times G\begin{array}{c} 1,1\\ 1,1 \end{array}\left[\frac{m_{SE}\Omega_{JE}}{\Omega_{SE}(S_{j}-1)}\gamma_{j}\left|\begin{array}{c} 1-k-m_{JE}(N_{JE}-1)\\ 0 \end{array}\right.\right]\frac{A}{2}\\ \times \sum_{k=0}^{N(\beta -1)}a_{k}{\left(\frac{\gamma }{\bar{\gamma_{RD}}}\right)}^{\frac{N\alpha +N+K}{4}}G_{1,3}^{2,1}\left[\frac{N\alpha \beta }{g\beta +\Omega^{\prime }}\sqrt{\frac{\theta \gamma }{\bar{\gamma_{RD}}}}\left|\begin{array}{l} k_{1}\\ k_{2} \end{array}\right.\right] \end{array}\right) \end{array}$$

where $H_{j}=\frac{\Gamma (n+1/2)x_{j}}{n!(n+1{)}^{2}[L_{n}^{\left(-1/2\right)}(x_{j}){]}^{2}}$, and $x_{j}$ is the jth root of the generalized Laguerre polynomial $L_{n}^{\left(-\frac{1}{2}\right)}(x)$.

## 5. Average Secrecy Capacity Analysis

The ASC is an important indicator used to evaluate the security performance of active eavesdropping,

(24) $${\bar{C}}_{S}=\int_{0}^{\infty }\frac{F_{\gamma_{SE}}(\gamma )}{1+\gamma }\left(1-F_{\gamma_{SRD}}(\gamma )\right)d\gamma$$

Substituting (11) and (17) into (24), followed by a few of the mathematical simplification operations mentioned above, results in the following expression:

(25) $$\begin{array}{l} {\bar{C}}_{S}=\sum_{p=0}^{N_{S}m_{SR}-1}\frac{1}{p!}{\left(\frac{m_{SR}}{\Omega_{SR}}\right)}^{p}\\ \times \left(\begin{array}{l} G\begin{array}{c} 2,1\\ 1,2 \end{array}\left[\frac{m_{SR}}{\Omega_{SR}}\left|\begin{array}{c} -p\\ 0,-p \end{array}\right.\right]-\sum_{j=1}^{t}H_{j}\frac{\gamma_{j}^{p+0.5}}{1+\gamma_{j}}\exp\left[-\left(\frac{m_{SR}}{\Omega_{SR}}-1\right)\gamma_{j}\right]\\ \times \frac{A}{2}\sum_{k=0}^{N(\beta -1)}a_{k}{\left(\frac{\gamma }{\bar{\gamma_{RD}}}\right)}^{\frac{N\alpha +N+K}{4}}G_{1,3}^{2,1}\left[\frac{N\alpha \beta }{g\beta +\Omega^{\prime }}\sqrt{\frac{\theta \gamma }{\bar{\gamma_{RD}}}}\left|\begin{array}{l} k_{1}\\ k_{2} \end{array}\right.\right] \end{array}\right)\\ -\sum_{t=0}^{m_{SE}-1}\frac{1}{t!}{\left[\frac{m_{SE}}{\Omega_{SE}(S_{j}-1)}\right]}^{t}\frac{1}{\Gamma (m_{JE}(S_{j}-1))}\sum_{k=0}^{t}\left(\begin{array}{c} t\\ k \end{array}\right){\left(\frac{\Omega_{JE}}{m_{JE}}\right)}^{k}\\ \times \sum_{p=0}^{N_{S}m_{SR}-1}\frac{1}{p!}{\left(\frac{m_{SR}}{\Omega_{SR}}\right)}^{p}\sum_{j=1}^{t}H_{j}\frac{\gamma_{j}^{p+0.5}}{1+\gamma_{j}}\exp\left[-\left(\frac{m_{SR}\theta }{\Omega_{SR}}+\frac{m_{SE}}{\Omega_{SE}(S_{j}-1)}-1\right)\gamma_{j}\right]\\ \times G\begin{array}{c} 1,1\\ 1,1 \end{array}\left[\frac{m_{SE}\Omega_{JE}}{\Omega_{SE}(S_{j}-1)}\gamma_{j}\left|\begin{array}{c} 1-k-m_{JE}(S_{j}-1)\\ 0 \end{array}\right.\right]\\ \times \left(1-\frac{A}{2}\sum_{k=0}^{N(\beta -1)}a_{k}{\left(\frac{\gamma }{\bar{\gamma_{RD}}}\right)}^{\frac{N\alpha +N+K}{4}}G_{1,3}^{2,1}\left[\frac{N\alpha \beta }{g\beta +\Omega^{\prime }}\sqrt{\frac{\theta \gamma }{\bar{\gamma_{RD}}}}\left|\begin{array}{l} k_{1}\\ k_{2} \end{array}\right.\right]\right) \end{array}$$

## 6. Simulation Results and Analysis

In this section, the simulation results of the mixed RF/FSO system under the influence of various parameters are provided, and Monte Carlo simulations are also given to verify the accuracy of the numerical results. We assume that on the RF link $d_{SE}=$ $d_{SR}=d_{JE}=10\;m$, the FSO link distance is 1 km, the wavelength is 785 nm, the optical wavenumber k=2π/λ, the refractive index structure constant $C_{n}^{2}=10^{-11}\;m^{-2/3}$, the FSO link instantaneous SNR ${\bar{\gamma }}_{RD}=20\;dB$, the RF link instantaneous SNR $\lambda_{SR}=\;15\mathrm{dB}$, and the instantaneous SNR of the eavesdropping link $\lambda_{SE}=-10\;dB$, while the target secrecy rate $R_{S}=0.01\;nat/s$. The above parameters are adopted by considering the ground communication scenario on the atmospheric turbulence channel. Other parameters are ρ=0.8, η=0.8, $m_{SR}=m_{SE}=2$, $S_{j}=2$, $N_{R}=2\;and\;\lambda_{JE}=3\;dB$. Other relevant data are shown in Table 1. The following simulation parameters adopt the above values when no additional annotations are made. When calculating the generalized Laguerre orthogonal numerical integration method, j is set to 30 to make the series converge. In order to verify the validity of the analytical expressions, Monte Carlo simulation results are presented.

The above values we used for the simulation parameters are primarily based on reference [7], which provides detailed parameters for similar systems and conditions. These values were chosen to ensure that the simulation accurately reflects real-world system configurations and is consistent with prior research in the field. The numerical results are in good agreement with the simulation results, which verifies the accuracy of the proposed expression.

**Figure 3:**
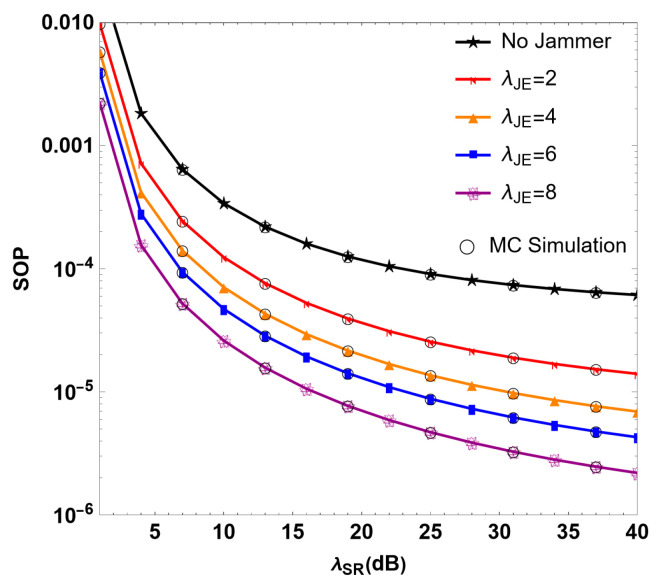
Simulation diagram of SOP under different SNR $\lambda_{JE}$ values of interference in the RF/FSO system.

**Figure 4:**
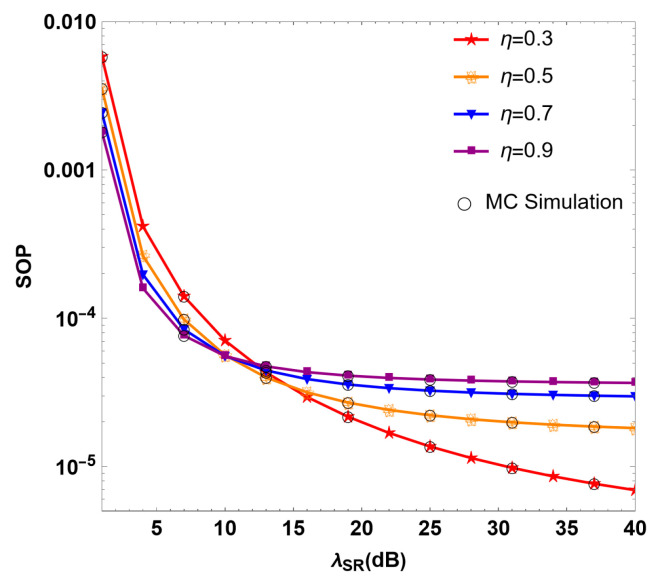
Simulation diagram of SOP under different energy conversion efficiency η values in the RF/FSO system.

**Figure 5:**
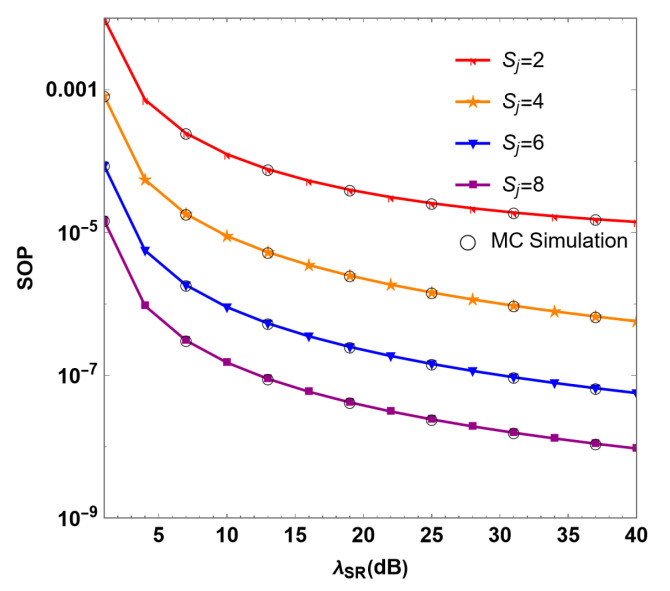
Simulation diagram of SOP under different numbers of interfering users $S_{j}$ in the RF/FSO system.

Figure 3 describes the relationship between the SOP and the instantaneous SNR $\lambda_{SR}$ of the RF link in the RF/FSO system when the untrusted relay interferes with interference in different SNR values $\lambda_{JE}$. The results indicate that with the increase in $\lambda_{SR}$, the SOP decreases gradually. When $\lambda_{SR}=30\;\mathrm{dB}$, in cases of $\lambda_{JE}=$ 2, 4, 6 and 8, the system SOP values are $1.09\;\times \;10^{-5}$, $9.78\;\times \;10^{-6}$, $6.20\;\times \;10^{-6}\;and\;3.26\;\times \;10^{-6}$. In the absence of the interference of interfering users, the SOP of the system is $7.38\;\times \;10^{-5}$. It can be observed that the SOP decreases significantly with the increase in $\lambda_{JE}$, which indicates that the interfering users can significantly improve the security performance of the system by sending interference signals to the untrusted relay. During the communication process, increasing the $\lambda_{JE}$ of interference can negatively impact the quality of the signal received by the untrusted relay. However, $\lambda_{JE}$ is limited by the transmitted power. Therefore, introducing the interference of interfering users and selecting a reasonable transmission power can effectively improve the security performance of the system. In the “error layer” shown in Figure 3, due to the randomness inside the communication system, under the condition of high SNR, the SOP will reach the minimum limit when the SNR increases to about 7 dB. Even if the SNR continues to increase, the value of SOP will not continue to decrease. The same is true of Figure 4 and Figure 5. At this time, the SNR is controlled at about 40 dB, and this can achieve the best system performance while saving costs.

Figure 4 describes the relationship between the SOP of the RF/FSO system and the $\lambda_{SR}$ of the RF link under different multi-user energy conversion efficiency values η. As the figure shows, the SOP of the system decreases with the increase in $\lambda_{SR}$ for different values of η. When $\lambda_{SR}<11\;dB$, the SOP of the system decreases with the increase in η. However, when $\lambda_{SR}>11\;dB$, the SOP gradually increases with the increase in η. It can be observed that in the case of $\lambda_{SR}<11\;dB$, the efficiency of the multi-user node in converting the received relay signal into DC can improve the security performance of the system. However, when $\lambda_{\mathrm{SR}}>11\;\mathrm{dB}$, the energy conversion efficiency increases, and the security performance degrades instead of improving. Therefore, when the channel gain from the sender to the relay is large enough, it is not necessary to send messages with maximum power. Although sending information at maximum power can improve the system’s security capability to a certain extent, it also has a positive impact on the communication performance of untrusted relays. At this time, the SNR is controlled at about 10 dB, which can achieve the best system performance while saving costs.

Figure 5 shows the relationship between the SOP of the mixed RF/FSO system and the $\lambda_{SR}$ of the RF link under different numbers of interfering users $S_{j}$. It can be seen from the figure that when $\lambda_{SR}\;=30\;\mathrm{dB}$ and the numbers of interferer antennas are $S_{j}\;=2,\;4,\;6,\;\mathrm{and}\;8$, the SOP values are $1.90\;\times \;10^{-5}$, $9.40\;\times \;10^{-7}$, $9.38\;\times \;10^{-8}\;and\;1.57\times \;10^{-8}$, respectively. As the number of interfering users increases, the SOP of the system decreases, and the security performance of the system is improved. Therefore, it can be inferred that the security performance of the system is significantly improved with the increase in the number of interfering users. This is because the increase in the number of interfering users degrades the communication performance of untrusted relay, which provides better security to the system. Thus, to achieve the same SOP, a scheme with a large number of interfering users has a lower transmission power compared to a scheme with no interfering users. This provides a good solution to the design of jammer equipment, and reduces the consumption at the transmitter side. At this time, the SNR is controlled at about 13 dB, which can achieve the best system performance while saving costs.

**Figure 6:**
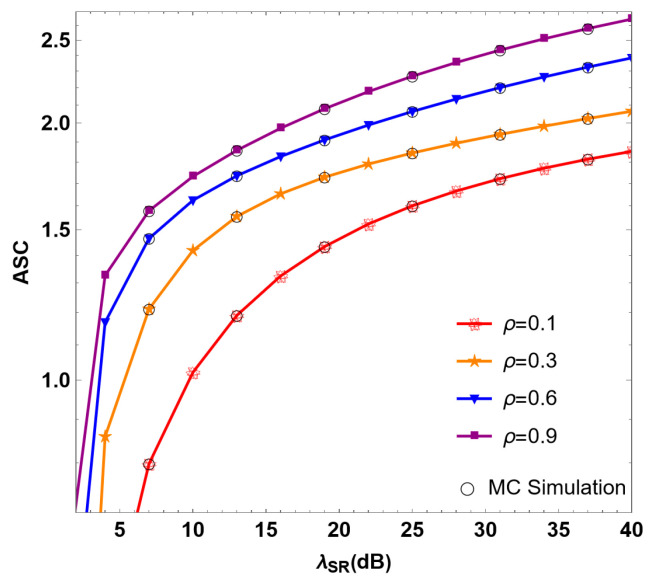
Simulation of ASC under different time distribution factors ρ in RF/FSO system.

Figure 6 shows the relationship between the ASC of the RF/FSO system and the $\lambda_{SR}$ of the RF link under different time distribution factors ρ for the multi-user scenario. When $\lambda_{SR}\;=30\;\mathrm{dB}$, in the case of ρ=0.1, 0.3, 0.6, and 0.9, the ASC values are 1.72, 1.94, 2.20, and 2.43, respectively. It can be determined that the ASC of the system increases with the increase in the value of ρ. As ρ is positively correlated with the effective energy obtained by the multi-user system, the larger the energy, the more energy is available to interfere with the transmitted interference signal stored and used by the user. This improves the interference effects of the interfering users on the untrusted relay, making the communication of the system more secure.

**Figure 7:**
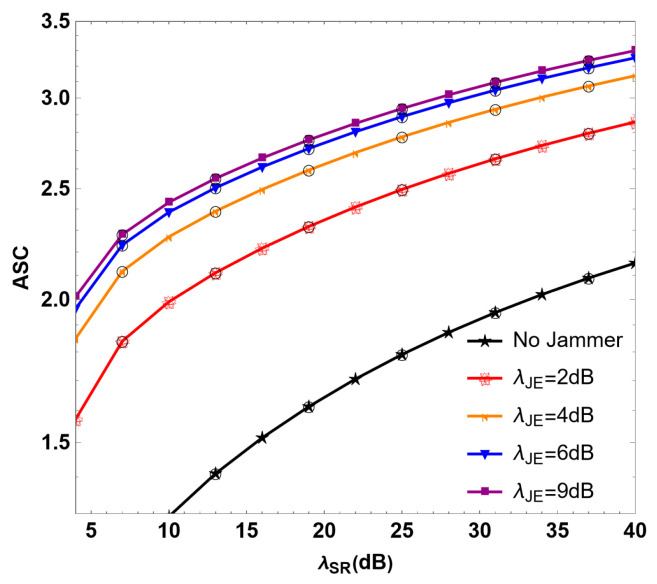
Simulation diagram of ASC under different SNR $\lambda_{JE}$ values of interference in the RF/FSO system.

Figure 7 describes the relationship between the ASC and $\lambda_{SR}$ of the RF link in the RF/FSO system under different values of $\lambda_{JE}$. When $\lambda_{SR}\;=30\;\mathrm{dB}$, the ASC values are 2.65, 2.93, 3.04 and 3.09f or $\lambda_{JE}=2,\;4,\;6,\;and\;9\;dB$, respectively. In the absence of multi-user interference, the ASC is 1.95. It can be seen that the ASC of the system increases to some extent with the increase in $\lambda_{JE}$. It further shows that the interfering users can improve the security of the system by sending interference signals to the untrusted relay, and increasing the transmitting power can improve the PLS of the system.

Compared to the results of Wang et al. [23], the mixed RF/FSO system presented in this paper demonstrates a more significant reduction in SOP when facing different numbers of interfering users. Notably, when the number of interfering users increases to 6 or 8, the SOP drops to an extremely low level, highlighting the system’s stronger anti-interference capability and enhanced security. Additionally, by introducing a mechanism for the random allocation of user interference, the system achieves a substantial improvement in secrecy capacity. Under higher interference strength $\lambda_{JE}$, the system exhibits greater gains and improved secrecy performance, showcasing the efficient utilization of interference resources and superior system capabilities.

## 7. Conclusions

In this paper, the security performance of a mixed RF/FSO system based on SWIPT multi-user interference was studied. The safety outage probability (SOP) and average security capacity (ASC) of the system were analyzed by theoretical derivation and simulation, and the validity of the derived expressions was verified by the Monte Carlo method. The effects of the average signal–interference noise ratio of the interfering users, the number of interfering users, the time distribution factor and the energy conversion efficiency on the system’s safety performance were studied. In this study, it is assumed that the CSI of all links in the system is in a perfect state. However, in order to be closer to the actual situation, it can be further considered that the channel state information of all links in the system is not perfect. Therefore, in the subsequent research work, it is very meaningful to study the impact of an imperfect noise ratio on the physical layer security performance of the CSI system when it increases the interference effect on untrusted relays. The simulation results show that with the increase in the instantaneous signal-to-noise ratio of the RF link, the system SOP decreases and the ASC increases. When the signal–interference noise ratio of the interfering multi-user increased, the SOP of the system was significantly decreased, and the ASC increased gradually. This indicates that the interference effect on untrusted relay could be enhanced by increasing the SINR, which improves the security performance of the system. When the number of interfering users increased here, the SOP of the system decreased considerably. For the same value of SOP, the transmitter power could be reduced by increasing the number of interfering users, which could provide an effective scheme for the jammer equipment to reduce the energy consumption at the transmitter end. The SWIPT technology was used to adjust the time distribution factor to increase the time received by the energy collector, which strengthened the interference signal transmitted by the interfering multi-user and improved the security of the system. On the other hand, when the RF link’s instantaneous SNR was low, the increase in energy conversion efficiency enabled the energy collector to store more energy, enhanced the interference signal during signal transmission, rendered the collection process controllable, and enhanced the security capability of the system. In conclusion, the safety performance of the RF/FSO system was significantly improved under the action of interfering user jamming based on SWIPT, which could provide a good theoretical basis for engineering implementation.

## Figures and Tables

**Table 1:** Selection of simulation parameters for the mixed RF/FSO system.

System Parameters	Symbol	Value	System Parameters	Symbol	Value
Laser wavelength	λ	785 nm	Link distance per hop	d	10 m
Weak turbulence positive parameter	a	2.33	Weak turbulence fading parameters	b	4.53
Medium turbulence positive parameter	a	1.83	Medium turbulence fading parameters	b	3.94
Strong turbulence positive parameter	a	1.43	Strong turbulence fading parameters	b	3.53
Electro-optic conversion efficiency	η	0.8	Target secrecy rate	$R_{S}$	0.01 nat/s
Atmospheric refractive index structure constant	$C_{n}^{2}$	$10^{-11}\;m^{-2/3}$	Energy conversion efficiency	η	0.8
Time switching factor	ρ	0.8	The instantaneous SNR of the FSO link	$\gamma_{RD}$	20 dB
The instantaneous SNR of the RF link	$\gamma_{SR}$	15 dB	Number of interfering antennas	$N_{J}$	2

## Data Availability

Data are contained within the article.
